# Advancing Rural Palliative Dementia Care: An Updated Scoping Review

**DOI:** 10.1093/geroni/igaf122.418

**Published:** 2025-12-31

**Authors:** Jing Wang, Christine Ritchie, Kirsten Corazzini

**Affiliations:** University of New Hampshire, Durham, New Hampshire, United States; Massachusetts General Hospital, Boston, Massachusetts, United States; University of New Hampshire, Durham, New Hampshire, United States

## Abstract

Palliative and end-of-life care for people living with dementia in rural areas remains a significant challenge due to geographic isolation, specialized workforce shortages, financial barriers, and gaps in culturally sensitive care models. This study updates Elliot et al.’s scoping review by assessing recent evidence on rural palliative dementia care to identify stakeholders’ needs and explore emerging approaches and their impact. Following Arksey and O’Malley’s scoping review framework, we conducted a systematic search of PubMed, EMBASE, CINAHL, PsycINFO, Web of Science, Cochrane Library, and ProQuest for studies published from November 2018 to the present. A total of 2,592 studies were identified, with 234 full-text articles screened and 48 studies included. Compared to the previous review, which primarily identified systemic barriers and policy gaps, this update reveals a growing focus on technology-enabled interventions such as telehealth, remote monitoring, and virtual peer support programs. However, digital literacy and infrastructure limitations hinder widespread implementation. Studies continue to highlight persistent challenges in accessing dementia-specific palliative services due to provider shortages, transportation barriers, and financial constraints. Caregiver burden remains a major concern, with rural caregivers facing increased emotional and physical strain due to limited respite care and professional support. Additionally, there is a critical need for community-driven solutions to improve rural dementia palliative care. Despite progress, critical gaps remain in integrating sustainable telehealth, expanding caregiver support, and developing community-responsive care models. Future research should prioritize leveraging community networks, home-based care models, and digital health innovations to enhance equity in rural dementia care.

